# Vector activity and propagule size affect dispersal potential by vertebrates

**DOI:** 10.1007/s00442-012-2293-0

**Published:** 2012-03-15

**Authors:** Casper H. A. van Leeuwen, Marthe L. Tollenaar, Marcel Klaassen

**Affiliations:** 1 Aquatic Ecology, Netherlands Institute of Ecology, Royal Netherlands Academy of Arts and Sciences (NIOO-KNAW), P.O. Box 50, 6700 AB Wageningen, The Netherlands; 2 Animal Ecology and Ecophysiology, Institute for Water and Wetland Research, Radboud University Nijmegen, Heyendaalseweg 135, 6525 AJ Nijmegen, The Netherlands; 3 Aquatic Ecology and Environmental Biology, Institute for Water and Wetland Research, Radboud University Nijmegen, Heyendaalseweg 135, 6525 AJ, Nijmegen, The Netherlands; 4 Landscape Ecology, Utrecht University, H.R. Kruyt Building, Padualaan 8, 3584 CH Utrecht, The Netherlands; 5 Centre for Integrative Ecology, School of Life and Environmental Sciences, Deakin University, Waurn Ponds Campus, Geelong, VIC 3217 Australia; 6 NIOO-KNAW, Droevendaalsesteeg 10, 6708 PB Wageningen, The Netherlands; 7 Animal Ecology, Netherlands Institute of Ecology, Royal Netherlands Academy of Arts and Sciences (NIOO-KNAW), P.O. Box 50, 6700 AB Wageningen, The Netherlands

**Keywords:** Digestion, Endozoochory, Metabolic rate, Physiology, Retention time

## Abstract

Many small organisms in various life stages can be transported in the digestive system of larger vertebrates, a process known as endozoochory. Potential dispersal distances of these “propagules” are generally calculated after monitoring retrieval in experiments with resting vector animals. We argue that vectors in natural situations will be actively moving during effective transport rather than resting. We here test for the first time how physical activity of a vector animal might affect its dispersal efficiency. We compared digestive characteristics between *swimming*, *wading* (i.e. resting in water) and *isolation* (i.e. resting in a cage) mallards (*Anas platyrhynchos*). We fed plastic markers and aquatic gastropods, and monitored retrieval and survival of these propagules in the droppings over 24 h. Over a period of 5 h of *swimming*, mallards excreted 1.5 times more markers than when *wading* and 2.3 times more markers than *isolation* birds, the pattern being reversed over the subsequent period of monitoring where all birds were resting. Retention times of markers were shortened for approximately 1 h for *swimming*, and 0.5 h for *wading* birds. Shorter retention times imply higher survival of propagules at increased vector activity. However, digestive intensity measured directly by retrieval of snail shells was not a straightforward function of level of activity. Increased marker size had a negative effect on discharge rate. Our experiment indicates that previous estimates of propagule dispersal distances based on resting animals are overestimated, while propagule survival seems underestimated. These findings have implications for the dispersal of invasive species, meta-population structures and long distance colonization events.

**Electronic supplementary material:**

The online version of this article (doi:10.1007/s00442-012-2293-0) contains supplementary material, which is available to authorized users.

## Introduction

Many small organisms can be transported alive in the digestive system of more mobile vertebrates, i.e. by endozoochory. The potential importance and generality of this process was recognized a long time ago (Darwin 1859; Ridley 1930). Mammals, such as bears, foxes and musk ox, forage on seeds and fruits, and defecate surviving seeds after travelling tens of kilometres across the landscape (e.g., Bruun et al. 2008; Koike et al. 2011). Many migratory animals, such as wildebeest, reindeer, fish, turtles, and numerous species of birds, can potentially transport seeds and invertebrates in various life stages (hereafter referred to as “propagules”) over even hundreds of kilometres (Liu et al. 2004; Anne Bråthen et al. 2007; Traveset et al. 2008; Brochet et al. 2009; Pollux 2011; Raulings et al. 2011).

This dispersal potential of propagules is often assessed experimentally. Captive animals, ranging from waterbirds to monkeys to foxes, are fed a known quantity of specific propagules, and kept in cages while feces are examined for retrieval of viable organisms (Varela and Bucher 2006; Spiegel and Nathan 2007; Brochet et al. 2010; Figuerola et al. 2010; Tsuji et al. 2010). This way, the survival of gut passage and the timing of retrieval is assessed and used to estimate expected and maximum dispersal distances. However, vector organisms will have to move to other locations to enable dispersal. None of the experiments to date have addressed the potential effects of the movement of vectors on their digestive performance and the resulting dispersal kernels (i.e. the function that describes the probability of dispersal to different distances). Movement, and high levels of activity in general, likely require reallocation of blood flow from the digestive system to muscle tissues and other organs supporting the activity (Brouns and Beckers 1993). Hence, retention times and propagule survival might be different for actively moving (and dispersing) vectors than for inactive animals in cages.

Previous experiments have been conducted in which smaller propagules are retrieved in higher numbers and are retained longer in the digestive system (e.g., DeVlaming and Proctor 1968; Soons et al. 2008; Figuerola et al. 2010). However, not all studies found similar effects of seed size (Varela and Bucher 2006; Wongsriphuek et al. 2008; Brochet et al. 2010). Most experiments compared passage of propagules that not only differ in size but inevitably also in other characteristics such as resistance to digestion and shape. Knowledge on how propagule size per se affects dispersal distance and how this might interact with the level of activity of the vector is still limited.

We here present the first experiment in which propagule dispersal is investigated in active animals. In the experiment, we also investigated the effect of propagule size. We compared digestive intensity and propagule retention times between mallards (*Anas platyrhynchos*) swimming in a flume tank and inactive controls. Swimming was expected to increase the metabolic rate of the birds, and hence affect their digestion. We used retrieval of aquatic snails [*Hydrobia* (*Peringia*) *ulvae*], previously found to survive digestion by ducks (Anders et al. 2009; Cadée 2011; Van Leeuwen et al. 2012), to measure changes in digestive intensity. The effect of propagule size was included by feeding plastic markers as surrogate propagules that differ in size, but are otherwise identical.

## Materials and methods

### Training and the flume tank

Sixteen adult mallards (*Anas platyrhynchos*) were trained twice a week to swim in water flowing at 0.7 m/s in an outdoor setup, starting May 2009 for 8 weeks. In late June, training was continued in an indoor flume tank until 12 mallards swam voluntarily and continuously for 5 h at a velocity of 1.11 m/s. The flume was oval-shaped, made of PVC and filled with tap water. Two silent outboard engines on 12-V batteries positioned at the start of either long side of the oval each produced a 214-N thrust, creating a near-laminar flow of 1.11 m/s (Online Resource Fig. A1). At the end of each long side, two rectangular cages (LWH: 0.72 × 0.46 × 0.10 m) kept the mallards in the flume tank. This allowed four mallards to be in the flume tank simultaneously. In the cages closest to the engines, a horizontal 12-mm mesh wire layer was placed 0.02 m below the water surface. Therefore, mallards in these cages could not swim but instead sat on the mesh wire in the same situation and water current as the swimming birds. Behind each set of two cages, droppings were retrieved in sieves with 1.5-mm mesh (the two mallards at the same side of the tank were fed different propagules during each experiment, allowing collection from two individuals in the same sieve). Control birds were individually housed in isolation cages constructed of 12-mm-thick wood (LWH: 0.54 × 0.46 × 0.48 m). The floor and part of the front of each cage was made of 12-mm mesh wire, and the cages were placed side by side. This allowed the birds to see their surroundings but not their conspecifics, and allowed us to collect their feces in a removable tray without disturbing the birds. Average air and water temperatures during the experiment were 23 and 18°C, respectively.

### The experiment

Propagule retrieval was compared between mallards subjected to three different treatments: *isolation*, *swimming* and *wading*; (1) two *isolation* birds were kept in the isolation cages for 24 h, (2) two *swimming* birds were swimming continuously for 5 h at 1.11 m/s in the flume tank, after which they were also transported to isolation cages and kept there for an additional 19 h and (3) two *wading* birds sat on the mesh wire in the water in a flume tank for 5 h, and were thereafter transported to isolation cages. After 24 h, all birds returned to the outdoor aviary. The total experiment took place over 18 days (11–28 July 2009) with 12 birds and three treatments in a random block design. Each experimental day, six birds performed trials simultaneously, and two consecutive experimental days were followed by one resting day. Each of the 12 individuals was therefore used once every 3 days in an experiment. We fed two propagule types; therefore each bird was involved in each treatment twice (12 birds, three treatments, two propagule types, totalling 72 trials in 12 experimental days with 6 rest days). During the experiment, all birds had access to freshwater ad libitum but not to food, to resemble the situation of travelling.

At the start of each experiment, all mallards were weighed and fed either of the two propagule types: round plastic markers (150 Polyoxymethylene balls, POM kogels; DIT Holland, Hilvarenbeek; 50 × 2 mm, 50 × 3 mm, 50 × 4 mm diameter) or live aquatic snails (300 *Hydrobia* (*P.*) *ulvae*, L: 4.3 ± 0.5 mm, W: 2.0 ± 0.2 mm, *n* = 100, mean ± SD, randomly selected and measured to the nearest 0.1 mm with callipers). Aquatic snails are part of the regular diet of mallards (Swanson et al. 1985; Gruenhagen and Fredrickson 1990; Baldwin and Lovvorn 1994; Rodrigues et al. 2002), and *Hydrobia* (*P.*) *ulvae* is a common marine snail with an operculum, of which a small percentage can survive passage through duck guts (Anders et al. 2009; Cadée 2011; Van Leeuwen et al. 2012). This species also occurs in brackish environments, and is not affected by short-term exposure to freshwater (Fenchel 1975).

Designated propagules were divided into portions and surrounded by a 1- to 2-mm layer of dough (i.e. moisturized ground wheat seeds) that created six pill-shaped “pellets” to facilitate feeding. A known quantity of propagules could be fed within minutes to each mallard, while minimizing handling stress and allowing exact determination of the time between ingestion and retrieval of propagules. The pellets did not affect the snails, as 100% of snails in control pellets (*n* = 50 per pellet, two pellets tested) survived at least 4 h.

Droppings were collected hourly until 12 h after ingestion, and once after 24 h. Retrieved plastic markers were sorted by size and counted, while snails were categorized into intact shells, fragments of shells, or viable snails. Intact shells were shells that did not show visible damage, and were measured for length and width with callipers. Broken shells and shell parts were counted as fragments. Viability of all intact shells was checked immediately by returning the snails to seawater and looking for movement or retraction reactions after touch under the microscope. In case of uncertainty, survival was checked every 4 h up to 48 h after excretion.

### Statistical analyses

The number of markers retrieved per trial and sampling interval followed a (overdispersed) Poisson distribution (based on normality of the residuals of the model) and was analyzed using repeated measures generalized mixed-effects models with Poisson error distribution, log-link function and random slope (Table 1). Treatment (*swim*, *wade* or *isolation*) was set as the fixed factor, with *swimming* as reference level to compare to both *isolation* and *wading* ducks. Retention time over the first 12 h (1–12, log-transformed, included linearly and squared), marker size (2, 3, and 4 mm) and mallard body mass at the start of each experimental day were set as covariates after centering (Raudenbush and Bryk 2002). Interactions initially included in the model were treatment:marker size, treatment:retention time, treatment:retention time^2^, marker size:retention time, and marker size:retention time^2^. Initial model AIC was 2,915, which lowered to 2,912 by removing insignificant treatment:marker size. Further removal of interactions lowered the AIC by <2, so these models were considered equivalent and no further interactions were removed.

**Table 1:** Results of the generalized mixed model for the chance of retrieval of markers from mallards (*Anas platyrhynchos*) including retention time

	St. coef	*z* value	Pr(>|*z*|)
(Intercept)	0.58	11.4	**<0.001**
Treatment *wade* (contrast *swim*)	−0.098	−1.4	0.17
Treatment *isolation* (contrast *swim*)	−0.099	−1.4	0.17
Retention time	−0.57	−5.0	**<0.001**
Retention time^2^	−1.2	−7.7	**<0.001**
Marker size	−0.26	−4.4	**<0.001**
Mallard body mass	0.022	0.51	0.61
Treatment *wade*:retention time	0.25	1.8	0.07
Treatment *isolation*:retention time	0.43	3.0	**<0.01**
Treatment *wade*:retention time^2^	0.34	1.5	0.14
Treatment *isolation*:retention time^2^	0.34	1.5	0.14
Marker size:retention time	0.15	1.3	0.20
Marker size:retention time^2^	0.41	2.2	**<0.05**

*Swimming* birds were set as reference level of treatment. Significant effects are in *bold*. Standardized coefficients indicate the relative contribution of the different factors to the model (Gelman 2008). The standard deviations for the random slopes of retention times were 0.18, i.e. 95% of the retention time slopes varied between −0.92 and −0.21 (Schielzeth and Forstmeier 2009). The repeatability for the intercept of random factor mallard was 3.4%, and additive overdispersion 0.50

Additive overdispersion was modeled by adding an extra random factor according to Nakagawa and Schielzeth (2010), i.e. overdispersion is absorbed by this added term, consisting of a random variable with a random level for each observation. To correct for possible differences between mallards in the intercept, individual mallard was taken as random factor. Retention time was included as random slope for this random factor, to account for the possibility that individual mallards could differ not only in mean number of markers excreted (which is indicated by the random intercept) but also in pattern of excretion over time (indicated by the random slope of each mallard) (Schielzeth and Forstmeier 2009). Model output was consistent when calculated with or without combinations of covariates as random slopes. Because the linear component of retention time was considered the most relevant covariate involved in significant interactions in the final model, we present output with only retention time included as random slope for random factor mallard.

The number of intact snails or markers retrieved during different phases of the experiments all followed Poisson distributions. Differences of retrieval between treatments were therefore compared in repeated measures generalized mixed-effects models with Poisson error distribution and log-link function. As dependent variables, we used either the total number of snails retrieved intact during the 5-h active phase, or the total retrieved during the subsequent 6- to 24-h inactive interval. For the markers, we analysed their total number retrieved over 24 h only, since their retrieval over the first 12 h was already addressed in the GLM including the more detailed retention time analysis enabled by the more frequent retrieval of markers than snails. In the three similar models, treatment was included as fixed factor (*swimming* as intercept) and centered mallard body mass as covariate. Individual mallard was taken as random factor. A potential size difference between ingested and excreted snails was tested using a Student’s *t* test. All calculations were performed using package lme4 in R for statistics (R Development Core Team 2011).

## Results

### Timing of marker retrieval

**Fig. 1:**
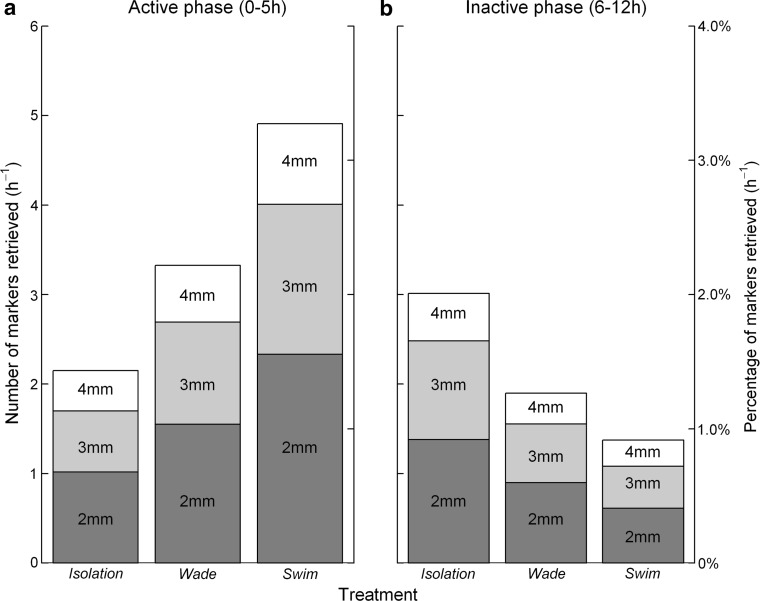
The mean number of 2-, 3-, and 4-mm markers retrieved per hour (*left* *y*-axes) and the percentage retrieved per hour (*right y*-axes) from mallards (*Anas platyrhynchos*) in isolation, wading, or swimming treatment during **a** the active phase (i.e. the first 5 h of the experiment where wading and swimming birds were in the flume tank) and **b** the inactive phase (6–12 h after the active phase), *n* = 12 individuals per treatment group. The significant interaction between wade and swim treatments as found by the GLM in Table 1 is visible

During the first 5 h of the experiment, in which the *swimming* birds were active, marker excretion in this group was increased compared to the *isolation* and *wading* birds. *Swimming* birds excreted 2.3 times more markers than *isolation*, and 1.5 times more than *wading* birds (Fig. 1a). After 5 h, when all birds were placed in the isolation cages, the *swimming* birds contrastingly excreted less than the *isolation* and *wading* birds. *Swimming* birds excreted 2.2 times fewer markers than birds in *isolation*, and 1.4 times fewer markers than *wading* birds between 6 and 12 h after ingestion (Fig. 1b). This caused both linear and non-linear effects of retention time (Table 1). The curvilinear component of retention time described the retention time curve most clearly as indicated by its highest standardized coefficient (Table 1; the parabolic curve visualized in Online Resource Fig. A2). Treatment affected only the linear component of retention time, dominated by the differences during the initial 5 h of the experiment rather than the overall retrieval over 12 or 24 h. This interaction (representing pattern of retrieval) was significantly different between *swimming* and *isolation* birds (Table 1), with *wading* birds intermediate but not significantly different from *swimming* birds (although *p* = 0.07; visualized in more detail in Online Resource Fig. A2). Average retention times of markers during the first 12 h were 5 h 20 min for *swimming*, 5 h 55 min for *wading* and 6 h 30 min for *isolation*. Total marker retrieval over 24 h was on average (±SD) still slightly higher for *swimming* birds (77.5 ± 27.0), compared to *wading* (68.9 ± 24.2) and *isolation* (70.5 ± 24.8). *Swimming* birds only differed significantly from *wading* birds, and marginally from *isolation* birds (GLM effect size *swim*–*wade* = −0.11, *z* = −2.3, *p* < 0.05; GLM effect size *swim*–*isolation* = −0.09, *z* = −1.84, *p* = 0.07). This implies that birds that had been *swimming* retained the fewest markers for longer than 24 h.

### Digestive intensity during exercise

**Fig. 2:**
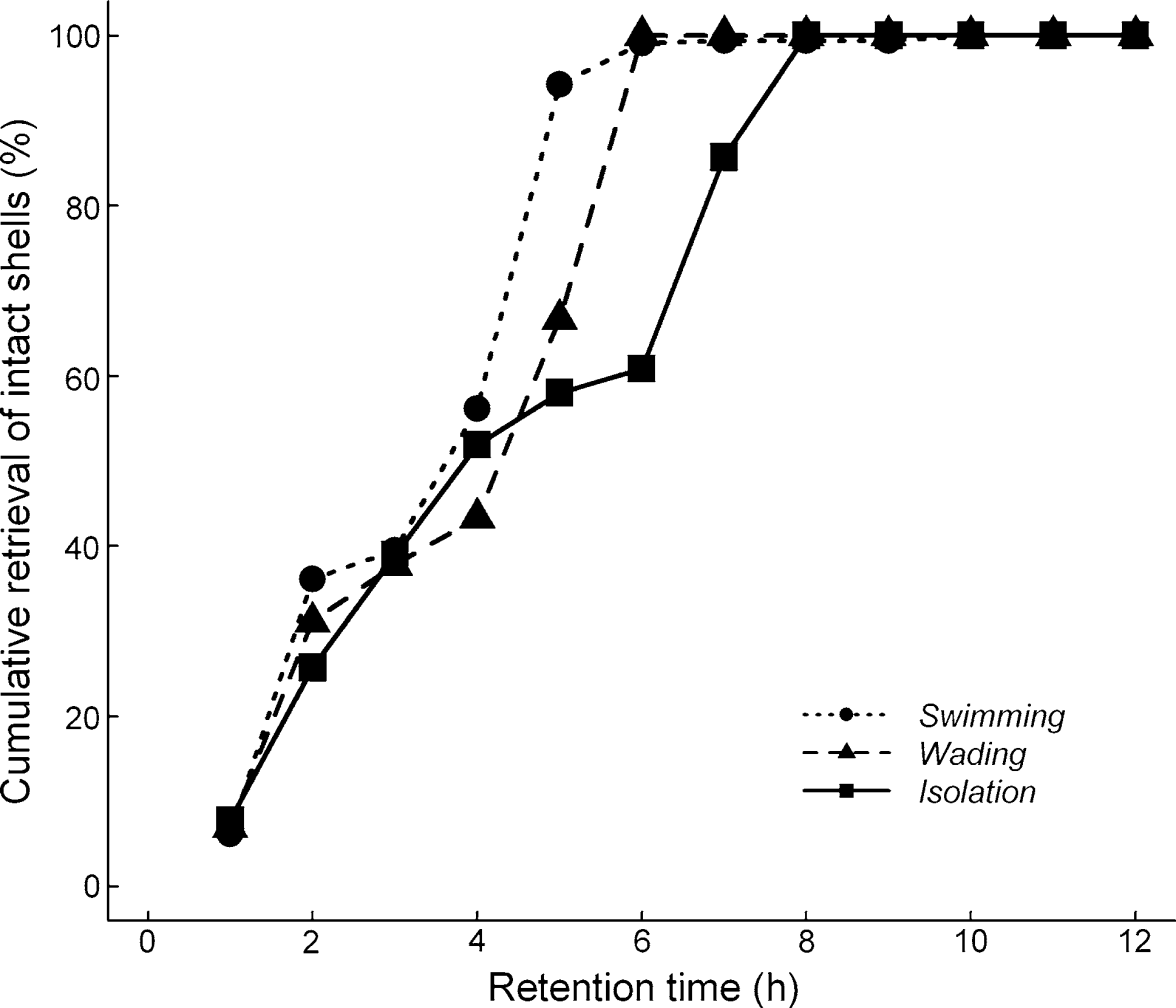
Cumulative percent of intact snail shells retrieved from mallards swimming, wading, or in isolation after 1–12 h propagule retention

The number of viable snails retrieved was too low to compare between treatments, but viable snails were retrieved from all three treatment groups (Online Resource Table A1). The last viable snail was retrieved after 7 h. The number of excreted intact shells, representing digestive intensity, was fewer for *wading* birds than for *swimming* birds over the active period (5 h) (average (±SD) for *wading* 1.6 ± 3.6; for *swimming* 4.8 ± 12.2; GLM effect size = −1.0, *z* = −3.7, *p* < 0.001). However, *isolation* birds did not differ from *swimming* birds (*isolation* 3.8 ± 5.6, GLM effect size = −0.34, *z* = −1.6, *p* = 0.10). After removal of all birds from the flume tank after 5 h there was no longer an effect of treatment in the 6- to 24-h interval (average ± SD of the number of excreted intact shells), although the analysis indicates a trend towards less excretion by *swimming* birds, intermediate excretion for *wading* and most excretion for *isolation* birds (*swimming* 1.0 ± 3.2, *wading* 1.75 ± 5.2, *isolation* 1.9 ± 4.9, *wading* effect size 0.47, *z* = 1.2, *p* = 0.22, *isolation* effect size = 0.65, *z* = 1.8, *p* = 0.07). This pattern is supported by a relatively fast retrieval pattern of intact shells from *swimming* birds, intermediate for *wading* and relatively slowest retrieval from *isolation* birds (Fig. 2). The total number of intact snails recovered after 12 h was 70 for swimming, 40 for wading, and 68 for control birds in isolation. No intact snails were retrieved after more than 12 h.

### Propagule size

Markers of 2 mm were retrieved 1.6 times more than 3-mm markers and 2.6-times more than 4-mm markers during the 24 h of the experiment, which was consistent over treatments (Fig. 1; Table 1; significant marker size but no interaction with treatment). The non-linear retrieval patterns of markers varied with marker size (see interaction, Table 1), but the cumulative release pattern of differently sized markers was similar over time (Online Resource Fig. A3). Consistent with the effect of plastic marker size, the average length of excreted snails (3.9 mm ± 0.04 SE, *n* = 178) was smaller than the average length ingested (4.3 mm ± 0.06 SE, *n* = 100, *t* = 5.98, *p* < 0.001).

## Discussion

### Activity affects propagule retention

Physical activity of mallards was found to modulate retention of ingested propagules in their digestive system. By inducing swimming in a flume tank, propagule excretion increased in comparison to inactive birds in dry cages or in the water. The metabolic rate of swimming mallards likely increased to more than four times that of birds resting in water (wading) (Prange and Schmidt-Nielsen 1970). The higher thermal conductivity of the wading birds in water might have increased their metabolic rate by as much as 25–30% compared to isolation birds (Prange and Schmidt-Nielsen 1970; Richman and Lovvorn 2011). We therefore suggest that the increased excretion of plastic markers is associated with an increase of metabolic rate. This corresponds to the intermediate number of markers excreted by wading birds and the fact that marker excretion of active animals was only higher during the actual active phase of the experiment, and again reduced after the activity stopped. Digestive rate is likely a flexible and rapidly adjustable process influenced by the metabolic rate of vectors.

Wading birds were included as a treatment because factors such as the ability to see conspecifics or the increase of thermal heat loss in water might have caused differences between isolation and swimming birds. The isolation birds allow comparison to the general situation of birds in previous endozoochory experiments, since most experiments with waterbirds to date have monitored retention times of inactive birds in such small and dry cages (Online Resource Table A2). Indeed, the wading birds with an increased thermal conductivity to water of only 18°C, excreted more makers than isolation birds. Such cage-effects by themselves can therefore already change experimental results.

Comparing swimming and wading birds to assess the effect of activity alone resulted in a 50% increase of propagule excretion during activity. Propagule retention times in experiments with inactive animals are therefore probably overestimated. That swimming birds also excreted the most propagules during the whole experiment over 24 h, although they were only active for 5 h of this time, indicates the potential for extreme long-distance transport may be lower than thus far inferred from experimental data in inactive birds. On the other hand, the experiments show that plastic markers were retained longer than 24 h in all treatments, suggesting a greater potential for long distance dispersal than previously anticipated in the literature for those propagules potentially able to survive such long retention.

### Digestive intensity

Propagule survival is known to decrease exponentially with increasing retention time (Charalambidou et al. 2003, 2005; Pollux et al. 2005). Hence, the shorter retention of propagules we found in active animals should result in higher viability, assuming digestive intensity is not influenced by activity. To compare digestive efficiencies between treatments directly, we included aquatic snails, *Hydrobia* (*P.*) *ulvae*, as propagules in our experiments. *H. ulvae* is an operculated snail that can close its shell and survive digestion by waterbirds (Anders et al. 2009; Cadée 2011; Van Leeuwen et al. 2012). Because of low retrieval of viable snails, we used retrieval of intact snail shells as a proxy for digestive intensity. As expected, the retrieval of snail shells in all treatments decreased with longer retention in birds (Online Resource Table A1).

We expected that activity would increase the blood flow to the birds’ lungs and muscles involved in physical activity, which would reduce the potential to allocate blood to the digestive system (Brouns and Beckers 1993). This could reduce digestive intensity. However, we found no clear evidence for this conjecture. Although swimming birds were less efficient in digesting snails than wading birds, the isolation birds were also less efficient than wading birds. The effect of activity on digestive intensity thereby remains inconclusive. Nevertheless, the shorter retention times of intact shells (Fig. 2) as well as markers (Fig. 1; Table 1) provides a mechanism that increases propagule survival. Shorter retention times for more active birds, without indications for increased digestive intensity, will result in higher survival of propagules but dispersal over shorter distances.

### Effect of propagule size on dispersal distance and retention

Propagule size is considered an important trait determining dispersal success. Smaller propagules are often retrieved in higher numbers and after longer retention in experiments (e.g., Traveset 1998; Soons et al. 2008; Figuerola et al. 2010). However, not all studies obtain similar results (Wongsriphuek et al. 2008; Brochet et al. 2010), and the propagules in most experiments did not only differ in size, but also inevitably in other aspects such as shape and structure (e.g., Mazer and Wheelwright 1993). In contrast, our experiment with indigestible markers that only differ in size indicates that larger propagules actually have potential for longer retention times than smaller propagules. This is similar to previous marker studies with other bird species (Grajal and Parra 1995; Figuerola and Green 2005). While larger organic propagules (that can be digested) are mostly retrieved at short retention times only, larger indigestible markers are on average excreted after longer retention. The indigestible markers were even occasionally retrieved from birds involved in a subsequent experimental treatment 3 days after initial ingestion (distinguishable by their yellow color). Since not all plastic markers were excreted within the 24 h of the experiments (see Online Resource Fig. A3), they were likely slowly released over the days following the feeding, perhaps after being retained as grit (Mateo et al. 2000). This supports the original suggestion that larger propagules are more likely to become trapped in the gizzard or other parts of the digestive system, and stay there for prolonged periods of time (DeVlaming and Proctor 1968). Because most large organic propagules are increasingly damaged during extremely long retention (up to days), they are mostly retrieved intact in experiments only after relatively short retention times. Our indestructible plastic markers indicate a potential for extreme long-distance dispersal by larger propagules (>2 mm) in case of sufficient resistance to digestion. Overall, smaller propagules (<2 mm) will have shorter exposure to digestive damage by passing the digestive system faster, and therefore have a higher success rate for dispersal. This is indeed what we observed for the aquatic snails. Smaller propagules will be quantitatively more dispersed but at shorter distances. Larger propagules might be transported over extremely long distances, but only if they can survive such long retention. Given the generally small size of propagules retrieved in waterbird droppings (e.g., Charalambidou and Santamaría 2005; Frisch et al. 2007), large propagules with this potential may be scarce.

### Implications

Our experiment involved waterbirds, chosen because of their suggested high importance as passive dispersal vectors (Bilton et al. 2001; Figuerola and Green 2002; Green and Figuerola 2005). In previous studies with waterbirds that assessed retention times of propagules experimentally, inactive animals had cage sizes varying between 3.0 × 3.0 m (L × W) and as small as 0.20 × 0.20 × 0.30 m (L × W × H), in which a duck can hardly move (Online Resource Table A2). Most frequently used cages measured 0.60 × 0.50 × 0.50 m, thus for comparison our isolation birds were kept in cages of similar size. However, the problem of artificially reduced activity in endozoochorous experiments goes beyond experiments with waterbirds. Retention times and dispersal distances of frugivorous terrestrial birds have also been inferred from birds held in small cages or even cotton bags (e.g., Spiegel and Nathan 2007; Lehouck et al. 2011). Feeding experiments with fish are performed in small tanks (e.g., Pollux et al. 2006; Anderson et al. 2009), and seed retention by mammals such as foxes, monkeys and elephants are inferred from animals retained in cages ranging from 1 to several square meters (e.g., Graae et al. 2004; Varela and Bucher 2006; Campos-Arceiz et al. 2008; Tsuji et al. 2010). Although all mentioned experiments make important contributions to our knowledge on dispersal, their estimated dispersal distances should be refined by correcting for potential activity of vectors. Thereby it should be borne in mind that different modes of transport may affect digestion differently. While swimming presumably increased the metabolic rate of the ducks fourfold, flying by the mallards would likely affect metabolic rate and digestive processes differently. Whether or not an even higher increase of metabolic rate (e.g., by flying) will further accelerate digestion or may instead reduce propagule excretion requires further research.

## Conclusion

The fact that active animals have shorter retention times and higher propagule survival rates implies that past estimates of long-distance dispersal potential using captive vertebrates may have overestimated dispersal distances, while underestimating propagule survival. These findings are of importance when constructing dispersal kernels to estimate dispersal distances of invasive species, assessing the capability of individuals to disperse across fragmented habitats, or estimating the colonization potential of rare species. Cage characteristics and circumstances (in water or on land) affect experimental outcomes. Experimentally monitoring propagule survival and retention times in flying birds, swimming fish, and moving mammals therefore provides interesting avenues for future research, but will require creative solutions for the practical issues involved in experiments with moving animals.

## Electronic supplementary material

Supplementary material 1 (PDF 293 kb)
