# Supplementary material for: Vector activity and propagule size affect dispersal potential by vertebrates
Source: Oecologia. 2012 Mar 15;170(1):101–9. doi: 10.1007/s00442-012-2293-0 (PMC3422460; doi:10.1007/s00442-012-2293-0)
Supplement: Supplementary file 1 — Supplementary material 1 (PDF 293 kb) [file 442_2012_2293_MOESM1_ESM.pdf]

Online Resource

Vector activity and propagule size affect dispersal potential by vertebrates

Casper H. A. van Leeuwen*, Marthe L. Tollenaar & Marcel Klaassen

** Corresponding author. Netherlands Institute of Ecology, Droevendaalsesteeg 10, 6708 PB Wageningen, The Netherlands, c.vanleeuwen@nioo.knaw.nl*

Oecologia 2012

Table A1: Sum of propagules ingested per treatment and retrieved from all 12 mallards per treatment.

			Markers			*Hydrobia (Peringia) ulvae*		
			2mm	3mm	4mm	Shells intact	Fragments	Living snails
	Ingested		600	600	600	3600		
Active (0-5h)	Retrieved	*Isolation*	122	82	54	45 (1.3%)	26 (0.7%)	8
		Wading	186	137	76	19 (0.5%)	65 (1.8%)	5
		Swimming	280	201	108	58 (1.6%)	88 (2.4%)	1
0-12h	Retrieved	*Isolation*	354	267	143	68 (1.8%)	110 (3.1%)	9
		Wading	337	246	134	40 (1.1%)	115 (3.2%)	5
		Swimming	382	280	157	70 (1.9%)	134 (3.7%)	1
12-24h	Retrieved	*Isolation*	23	31	28	0 (0%)	0 (0%)	0
		Wading	31	49	30	0 (0%)	0 (0%)	0
		Swimming	32	46	33	0 (0%)	0 (0%)	0

Table A2: Cage sizes for the sixteen most recent endozoochorous experiments with waterbirds. In all publications birds were individually kept and the most cages had floors of mesh wire (ranging from 9 to 12-mm). Publications are sorted starting with the largest cage size.

**Cage size, LxW(xH)**	**Publication**
3.00x3.00m	(Figuerola and Green 2005)
1.70x1.70 m	(Charalambidou et al. 2003c)
2.00x0.80x0.60m or 1.0x0.79x0.58m (with small water basin)	(Smits et al. 1989)
0.50x1.50m	(Bell 2000)
0.60×0.50×0.50m	(Santamaría et al. 2002; Charalambidou et al. 2003a; Charalambidou et al. 2003b; Charalambidou et al. 2005; Pollux et al. 2005; Soons et al. 2008; Brochet et al. 2010; Figuerola et al. 2010)
0.35x0.75x0.60	(Powers et al. 1978)
0.20x0.20x0.30m	(Wongsriphuek et al. 2008)
not mentioned	(Agami and Waisel 1986)
“pens”	(Mueller and Van der Valk 2002)

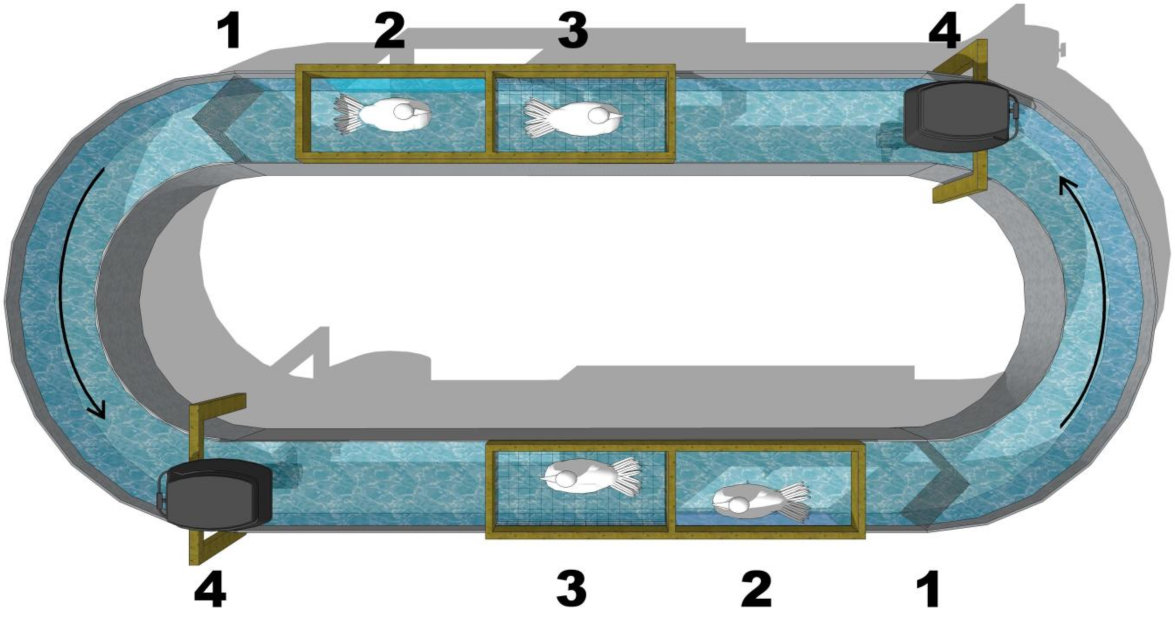

Figure A1: The indoor flume tank, with 1. Sieves for collecting droppings, 2. *Swimming* ducks, 3. *Wading* ducks sitting on mesh wire beneath the water surface preventing the mallards from swimming, 4. Electric outboard engines. The total construction was 4.80 m by 2.05 m, with tank width of 0.41 m and water depth of 0.37 m; the arrows indicate the direction of the current.

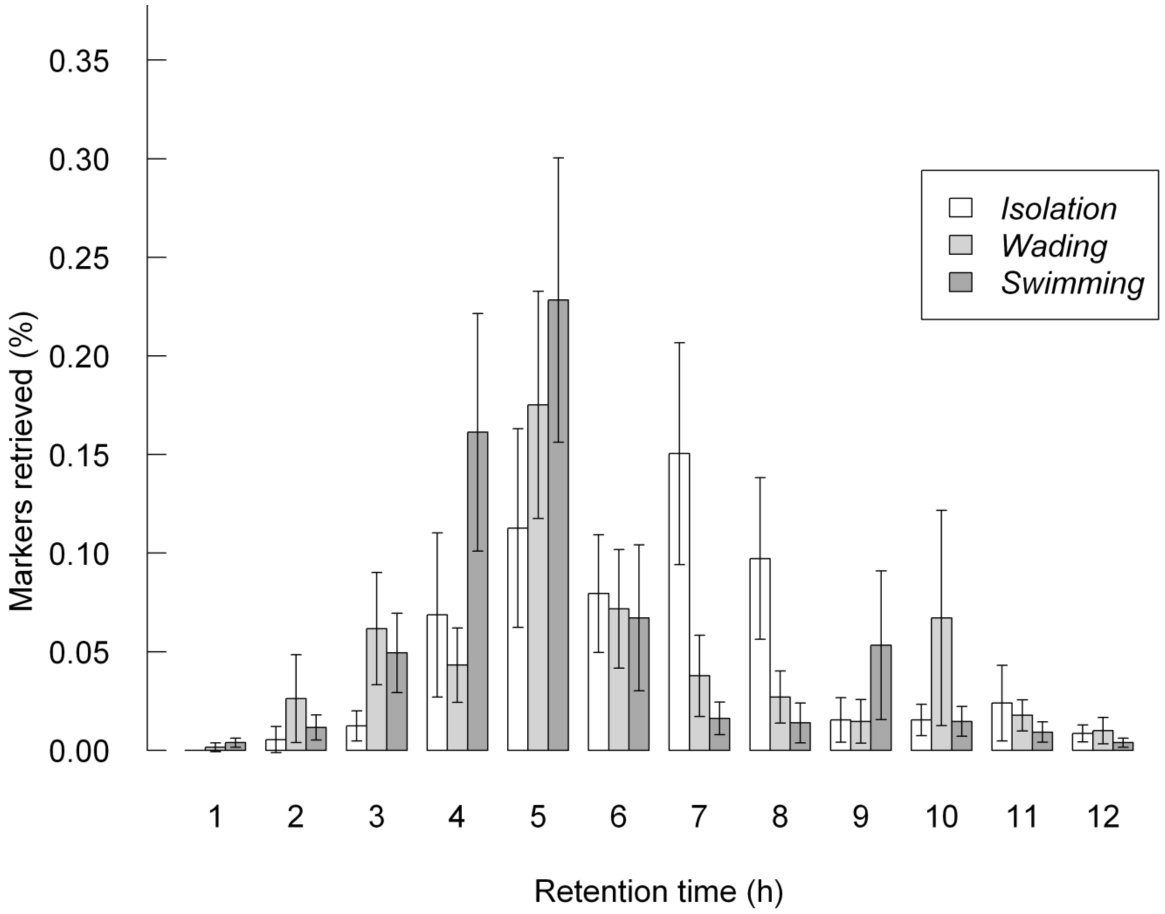

Figure A2: Retrieval of markers over time for the different treatments, average over all sizes (mean + SE).

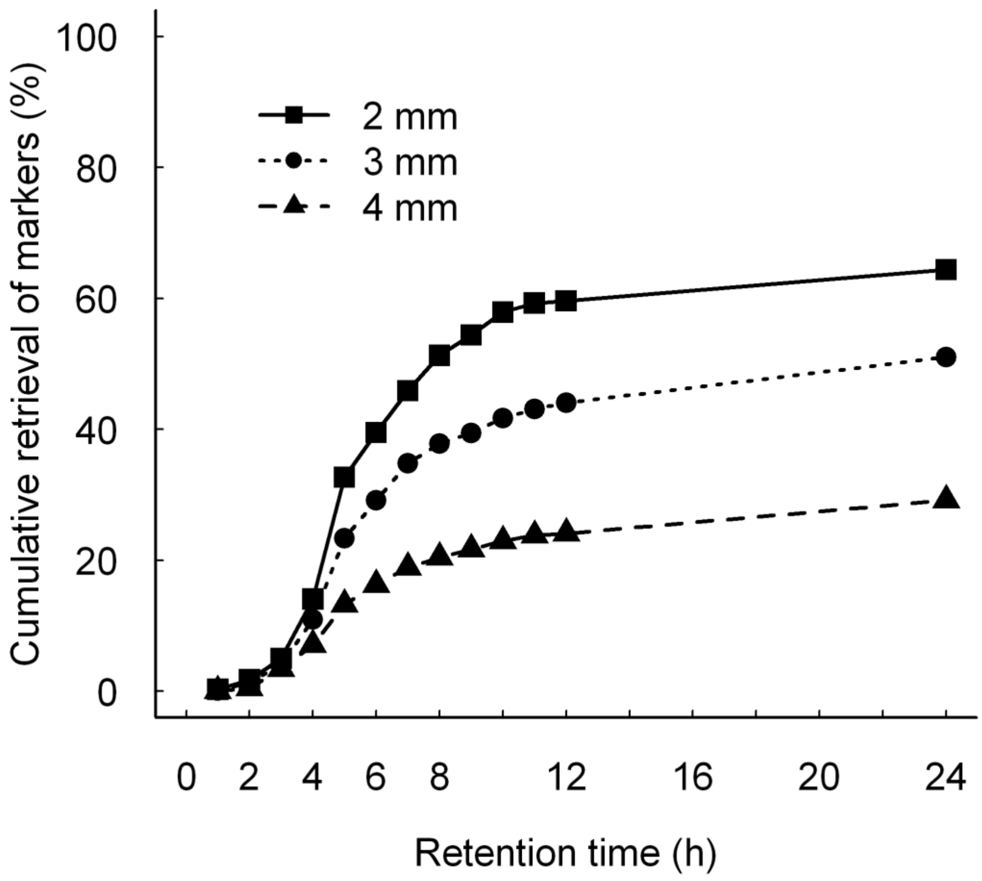

Figure A3: Cumulative retrieval of different sizes markers over time, averaged for all treatments and plotted as percentage of total ingested.

References

Agami M, Waisel Y (1986) The role of mallard ducks (*Anas platyrhynchos*) in distribution and germination of seeds of the submerged hydrophyte *Najas marina* L. Oecologia 68:473-475

Bell D (2000) The ecology of coexisting *Eleocharis* species. Phd-thesis

Brochet A-L, Guillemain M, Gauthier-Clerc M, Fritz H, Green AJ (2010) Endozoochory of Mediterranean aquatic plant seeds by teal after a period of desiccation: Determinants of seed survival and influence of retention time on germinability and viability. Aquat Bot 93:99-106

Charalambidou I, Ketelaars HAM, Santamaría L (2003a) Endozoochory by ducks: influence of developmental stage of *Bythotrephes* diapause eggs on dispersal probability. Divers Distrib 9:367-374

Charalambidou I, Santamaría L, Figuerola J (2003b) How far can the freshwater bryozoan *Cristatella mucedo* disperse in duck guts? Arch Hydrobiol 157:547-554

Charalambidou I, Santamaría L, Jansen C, Nolet BA (2005) Digestive plasticity in mallard ducks modulates dispersal probabilities of aquatic plants and crustaceans. Funct Ecol 19:513-519

Charalambidou I, Santamaría L, Langevoord O (2003c) Effect of ingestion by five avian dispersers on the retention time, retrieval and germination of *Ruppia maritima* seeds. Funct Ecol 17:747-753

Figuerola J, Charalambidou I, Santamaría L, Green AJ (2010) Internal dispersal of seeds by waterfowl: effect of seed size on gut passage time and germination patterns. Naturwissenschaften 97:555-565 doi:10.1007/s00114-010-0671-1

Figuerola J, Green AJ (2005) Effects of premigratory fasting on the potential for long distance dispersal of seeds by waterfowl: An experiment with marbled teal. Rev Ecol (Terre Vie) 60:283-287

Mueller MH, Van der Valk AG (2002) The potential role of ducks in wetland seed dispersal. Wetlands 22:170-178

Pollux BJA, Santamaría L, Ouborg NJ (2005) Differences in endozoochorous dispersal between aquatic plant species, with reference to plant population persistence in rivers. Freshw Biol 50:232-242

Powers KD, Noble RE, Chabreck RH (1978) Seed distribution by waterfowl in Southwestern Louisiana. J Wildl Manag 42:598-605

Santamaría L, Charalambidou I, Figuerola J, Green AJ (2002) Effect of passage through duck gut on germination of fennel pondweed seeds. Arch Hydrobiol 156:11-22

Smits AJM, Van Ruremonde R, Van der Velde G (1989) Seed dispersal of three nymphaeid macrophytes. Aquat Bot 35:167-180

Soons MB, Van der Vlugt C, Van Lith B, Heil GW, Klaassen M (2008) Small seed size increases the potential for dispersal of wetland plants by ducks. J Ecol 96:619-627 doi:10.1111/j.1365-2745.2008.01372.x

Wongsriphuek C, Dugger BD, Bartuszevige AM (2008) Dispersal of wetland plant seeds by mallards: Influence of gut passage on recovery, retention, and germination. Wetlands 28:290-299
